# Effect of *Flammulina velutipes* polysaccharides on the physicochemical properties of catfish surimi and myofibrillar protein oxidation during frozen storage

**DOI:** 10.3389/fnut.2023.1268580

**Published:** 2023-09-25

**Authors:** Liang Ling, Ying Liu, Xin Zhang, Tariq Aziz, Muhammad Shahzad, Manal Y. Sameeh, Ying Wang, Chunbo Cai, Yingchun Zhu

**Affiliations:** ^1^Shanxi Institute for Functional Food, Shanxi Agricultural University, Taiyuan, China; ^2^College of Food Science and Engineering, Shanxi Agricultural University, Taigu, China; ^3^Institute of Basic Medical Sciences, Khyber Medical University, Peshawar, Pakistan; ^4^Chemistry Department, Faculty of Applied Sciences, Al-Leith University College, Umm Al-Qura University, Makkah, Saudi Arabia; ^5^College of Animal Science and Technology, Shanxi Agricultural University, Taigu, China

**Keywords:** *Flammulina velutipes* polysaccharides, surimi, frozen storage, oxidation, cryoprotectant, myofibrillar protein

## Abstract

This study investigated the effect of *Flammulina velutipes* polysaccharides (FVPs) on the myofibrillar protein (MP) oxidation protein and physicochemical properties of catfish surimi during 75 days of frozen storage at −18°C. FVP was added to surimi at 1%, 1.5%, and 2%, respectively; the degree of MP oxidation and the physicochemical properties of the surimi were investigated, and the microstructure of the surimi was observed by scanning electron microscopy (SEM). The results showed that the carbonyl content and the thiobarbituric acid reactive substances (TBARS) in the FVP groups were lower than those in the CK group (the blank surimi). In comparison, the total sulfhydryl content, solubility, and Ca^2+^-ATPase activity were higher than those in the CK group after 75 days of storage. The addition of FVP significantly increased the water-holding capacity (WHC), gel strength, elastic modulus (G'), and loss modulus (G“) of surimi, and made the gel of surimi have stronger continuity and a denser structure. Therefore, FVP has a better cryoprotective effect on surimi. It improves the quality of surimi, decreases MP oxidation, and reduces lipid and water loss during frozen storage. The anti-freezing effect of FVP added at 2% was similar to that of commercial protectants (4% sucrose and 4% sorbitol).

## 1. Introduction

Surimi is a wet concentrate of myofibrillar proteins prepared from the muscles of deboned fish after removing blood, lipids, and other impurities using cold water (1). Frozen storage is one of the most effective ways to preserve surimi products at home and on a commercial scale (2). Frozen storage inhibits the growth and reproduction of microorganisms, reduces the activity of enzymes, and effectively extends the shelf life of surimi products. However, frozen storage can cause the quality of surimi to deteriorate, especially at prolonged storage times. This is due to the oxidation of proteins and fats by oxidative enzymes and pro-oxidants during frozen storage. The direct oxidation of aliphatic amino acids (3), the direct cleavage of peptide backbone chains (4), the Michael addition reaction of unsaturated aldehydes and proteins, and the non-enzymatic catalytic reaction of reducing sugars lead to the formation of carbonyl compounds (5). The conversion of sulfhydryl groups into disulfide bonds leads to a decrease in the sulfhydryl content (6), which is a sign of protein oxidative damage. Studies have reported protein degradation and lipid peroxidation as the two mechanisms responsible for the deterioration in quality and increased perishability when surimi is stored for a prolonged period. The structural changes in surimi proteins, especially myofibrillar protein (MP), occur either due to changes in the α-helical structure of surimi MP (7), an increase in the relative content of disordered curls (8–10), or conformational changes in the main chain (from ordered to disordered units). Moreover, surimi is rich in unsaturated fatty acids (11) and thus highly susceptible to oxidation during the freezing process. Lipid peroxidation in surimi produces many different aldehydes, ketones, and acids, resulting in the oxidation and decrease in gelation of MP, hence quality deterioration. To preserve and protect surimi quality during frozen storage, food industries commonly add 4% sucrose and sorbitol as commercial preservatives to inhibit the oxidation of MP (12). However, the sweetness and caloric content of these cryoprotectants are relatively high and not in line with the current concept of pursuing a healthy diet. Therefore, the search for cryoprotectants that are low in sweetness and caloric content has recently become a hot topic of research in food industries across the world. In this context, polysaccharides may offer a cost-effective, healthy, and safe alternative to traditional cryoprotectants for surimi preservation and maintaining quality during frozen storage.

Polysaccharides are natural polymers of aldose or ketose linked together by glycosidic bonds and found in plants, algae, bacteria, and animals. The structural unit in these molecules is monosaccharides that are linked by either α-1,4-glycosidic, β-1,4-glycosidic, or α-1,6-glycosidic bonds (13). Research evidence suggests that polysaccharides may have a cryoprotective effect on MP during the frozen storage of surimi. For example, Zhou et al. (14) reported that 8% seaweed sugar could effectively delay the denaturation process of MP in tilapia surimi and maintain Ca^2+^ATPase activity and MP solubility. Similarly, Nopianti et al. (15) reported that lactitol, alginate, and dextran exert an anti-freezing effect and prevent the denaturation of MP during frozen storage. Research on polysaccharides obtained from natural sources as cryoprotective agents is also underway.

*Flammulina velutipes* is known as the constructed mushroom and the hairy stalked money mushroom, which has a beautiful form, a fresh taste, and a high economic value (16). *Flammulina velutipes* is a rich source of polysaccharides, triterpenes, sterols, and vitamins with proven antioxidant, anti-inflammatory, immune-modulating, and antimicrobial activities. These beneficial activities have mainly been attributed to the presence of polysaccharides. *Flammulina velutipes* polysaccharide (FVP) is a water-soluble polysaccharide extracted from *Flammulina velutipes* mainly through hot water, enzyme, or ultrasound/microwave-assisted extraction methods (17–19). Zheng et al. (20) showed that the protein oxidation of freeze–thawed large yellow croaker was inhibited by FVP treatment, as demonstrated by the sulfhydryl group and intrinsic fluorescence intensity. Xie et al. (21) studied the effect of FVP on the quality of *Salmo salar* during frozen storage, and the results showed the best effect on protein oxidation by adding 0.15 mg/mL of FVP. In summary, FVP can effectively improve protein denaturation and potentially protein oxidation in the frozen food field. However, to date, the majority of the research has focused on the functional evaluation of FVP and paid less attention to its production and industrial use, thus greatly limiting the translational aspects of FVP research results.

In the current study, we have extracted the polysaccharide from the roots of *Flammulina velutipes*, which were often discarded due to their difficulty to swallow when consumed, and evaluated the effect of *Flammulina velutipes* polysaccharides on the physicochemical properties of catfish surimi and myofibrillar protein oxidation during frozen storage, with the ultimate goals being to gain deeper insights and potential for using FVP as a natural cryoprotectant in frozen surimi.

## 2. Materials and methods

### 2.1. Materials and reagents

*Flammulina velutipes* were purchased from the Edible Mushroom Research Centre, Shanxi Agricultural University (Jinzhong, Shanxi, China). A total of 102 live catfish (*C. gariepinus*) with an average weight of 1,250 ± 25 g and a length of 30 ± 2 cm were purchased from a local aquaculture farm at Taigu Taoyuanbao (Jinzhong, Shanxi, China). Sucrose and sorbitol were purchased from Shandong Dingsheng Chemical Co., Ltd. (Shandong, China), while the chemicals including ether, KCl, MgCl_2_, NaCl, HCl, glycine, and ethylenediaminetetraacetic acid were purchased from Chengdu Chron Chemical Co., Ltd.; urea, DTNB, Kormas Brilliant Blue, 1,4-Piperazinediethanesulfonic acid (PIPES), sodium pyrophosphate, and Na_2_HPO_4_ were purchased from Tianjin Kaitong Reagent Co., Ltd. (Tianjin, China); all the above reagents were of analytical grade.

### 2.2. Methodology

#### 2.2.1. Preparation of FVP

The FVP was extracted from the roots of *Flammulina velutipes* by ultrasound-assisted hot water extraction method. For this purpose, the roots of *Flammulina velutipes* were first washed and then dried at 60°C for 24 h until they reached a constant weight. The roots were then mechanically crushed into the powder using a commercially available grinder (model RT-02A, Rong Tsong Precision Technology Co., Ltd., Taiwan, China) and passed through a 200 mesh sieve. The powder was added to distilled water (1:25 w/v) and ultrasonicated at 70°C for 45 min followed by centrifugation at 8,000 rpm for 10 min at 4 °C by a high-speed freezing centrifuge (EPPENDERF China Co., Ltd., Beijing, China). The resultant solution was concentrated to one-fifth volume on a rotary evaporator (model RE 52A, Yarong Co., Ltd., Shanghai, China) at 55°C for 14 h and then added anhydrous ethanol at a ratio of 1: 3 (v/v) and left overnight at 4°C in order to obtain a precipitate. The precipitate was collected by centrifugation, placed at 78°C for pre-freezing, and dried in a vacuum freeze-drying machine (Ningbo Xinzhi Biotechnology, China) for 48 h. The FVPs were sent to Borealis Biotechnology Ltd. for testing, and the results showed that the FVP consisted of glucose (Glc), galactose (Gal), mannose (Man), fucose (Fuc), and xylose (Xyl) at a molar ratio of 55.5:25.9:10:5.5:2.

#### 2.2.2. Preparation of catfish surimi

The catfish were immediately transferred to the Meat Laboratory of Shanxi Agricultural University and kept in ice water (0–4°C) for 15 min to stun them. The head, gut, bones, and skin were removed, and a large piece of fish filet was obtained. The filet was washed two times with a 0.15% NaCl solution (0–4°C), dehydrated, and minced in a meat grinder. The minced fish were divided into five parts by adding FVP at 0%,1%, 1.5%, and 2% of fish meat (mass weight), respectively. As a positive control, no FVP or commercial protectant with 4% sucrose and 4% sorbitol was used, namely the CK group, 1% FVP group, 1.5% FVP group, 2% FVP group, and Y group. Five groups were chopped in a chopper mixer (Qingpu Food Packaging; China) for 3 min to obtain catfish surimi, and 20% ice water was added to maintain a low temperature during chopping. Finally, the five groups were packaged in polyethylene bags (75 millimeters in thickness, 20 x 30 cm in dimension) with an oxygen permeability of 0.8 mL m ^−2^ 24 h ^−1^ and a water vapor permeability of 2.4 g m ^−2^ 24 h ^−1^ at 23°C. The samples were frozen and stored at −18°C in a refrigerator (Beijing Fuyi Electric Appliance Co., Ltd., Beijing, China) and sampled at 15, 30, 45, 60, and 75 days for index measurements.

#### 2.2.3. Extraction and concentration measurement of surimi MP

The myofibrillar proteins (MPs) were extracted from catfish according to the Zhu et al. method (22) and stored at 4°C for backup. The concentration of protein was measured using the biuret method.

#### 2.2.4. Determination of total sulfhydryl content

The total sulfhydryl content was evaluated according to the method described by Cai et al. (23)

#### 2.2.5. Determination of the carbonyl content

The carbonyl content was analyzed according to the Levine et al. (24) method.

#### 2.2.6. Determination of MP solubility

A 5 mg/mL of MP solution was prepared with PIPES buffer solution (0.6 mol/L of NaCl, 0.02 mol/L of MgCl_2_, 0.1 mol/L of PIPES, and 0.01 mol/L of odium pyrophosphate, pH = 6.2). The solution was kept at 2°C for 4 h and shaken every 20 min during this period. After 4 h, the solution was centrifuged at 2°C at 2,000 g for 15 min, and 1 mL of the supernatant was taken to measure the MP concentration. MP solubility is calculated according to the following formula (1):


(1) $$\begin{array}{l} \text{MP solubility}=\frac{MP\;concentration\;of\;supernatant}{MP\;concentrations\;configured\;}\times 100 \end{array}$$

#### 2.2.7. Determination of Ca^2+^-ATPase activity

Ca^2+^-ATPase activity was measured by a commercially available Ca^2+^-ATPase activity assay kit (Solabao Biotechnology; China). The phosphorus content was quantified following the manufacturer's instructions, and the enzyme activity unit (U) was defined as the μmole number of inorganic phosphorus produced by a milligram of protein for 1 h at 25°C (μmol Pi/mg protein/h).

#### 2.2.8. Determination of water-holding capacity

The water-holding capacity (WHC) of surimi was evaluated using the centrifuge method. Briefly, a 2 g sample of surimi with two layers of Whatman filter paper was placed in a centrifuge tube and centrifuged at 2,000 g for 15 min at 4°C. The WHC is calculated as the percentage of the surimi weight after centrifugation divided by the surimi weight before centrifugation.

#### 2.2.9. Determination of color

The color of surimi was determined using the Xia et al. (25) method described previously. A colorimeter (CM-5 Konica Minolta Office System Co., Ltd., Tokyo, Japan) was used to determine the L^*^, a^*^, and b^*^ values. The whiteness of surimi was calculated using the following formula (2):


(2) $$\begin{array}{l} W=100-\left[\sqrt{{\left(100-L^{{}^{*}}\right)}^{2}+{a^{{}^{*}}}^{2}+{b^{{}^{*}}}^{2}}\right] \end{array}$$

where L^*^ represents lightness value, a^*^ denotes greenness/redness values, and b^*^ indicates blueness/yellowness values.

#### 2.2.10. pH measurement

Surimi (5 g) was homogenized for 30 s at a higher speed with 15 mL of distilled water. The pH of all samples was determined using an acidity meter (FE28, Metter Toledo, Shanghai, China).

#### 2.2.11. Determination of gel strength

The surimi gel was prepared according to the method of Yan et al. (26). Gel samples were analyzed with a spherical metal probe (P/5S) using a texture profile analyzer (SMATA.XTPlus, Stable Micro Systems, UK). Samples were analyzed at test speed (120 mm/min), trigger force (0.4 N), and compression distance (10 mm). Gel strength was calculated according to the formula (3)


(3) $$\begin{array}{l} Gelstrength\left(g\cdot mm\right)=breakagedistance\left(mm\right)\times breakageforce\left(g\right) \end{array}$$

#### 2.2.12. Determination of rheological properties

All rheological measurements were carried out using a rheometer (DHR-2, TA Instruments-Waters LLC, New Castle, DE, USA) equipped with a Peltier temperature control unit. Surimi samples frozen for 75 days were thawed and placed on the platform of the rheometer with a parallel plate pp50 test, spacing 1 mm, and temperature scanned in vibration mode within the linear viscoelastic zone, increasing the temperature from 20°C to 70°C at a ramp rate of 2°C/min, oscillation frequency set to 1 Hz, and strain set to 0.1%. The frequency sweep and temperature ramp measurements were carried out in the linear viscoelastic range (0.05% strain). The viscosity curves were determined under the controlled shear rate in a range of 1–10 s^−1^.

#### 2.2.13. Determination of TBARS values

TBARS values were analyzed by the method of Guan et al. (27).

#### 2.2.14. Microstructure

The microstructure of surimi gels was determined using scanning electron microscopy (SEM). Briefly, the surimi gels were prepared by cutting them into 3 mm × 3 mm × 2 mm cuboids. The cuboids were then fixed with 2.5% (v/v) glutaraldehyde in 0.2 M phosphate buffer solution (pH 7.2). The samples were rinsed for 1 h in distilled water and then dehydrated with serial concentrations in ethanol: 50%, 70%, 80%, 90%, and 100% (v/v). The dried samples were put on the aluminum stub using carbon tape, and the samples were coated with gold for approximately 60 s. The specimens were observed using a SEM (Quanta 450 SEM, FEI Company, Hillsboro, America).

### 2.3. Statistical analysis

Each experimental procedure was performed on three replicates. The data obtained were expressed as the mean values ± standard deviations and analyzed using SPSS version 22.0 (SPSS Inc., USA). One-way ANOVA was used to analyze all the data by DUNCAN's test with significance values of *P* < 0.05. All figures were designed using Origin Pro 9 (Origin Lab Inc., USA).

## 3. Results

### 3.1. Effect of FVP on the total sulfhydryl group content of surimi

**Figure 1:**
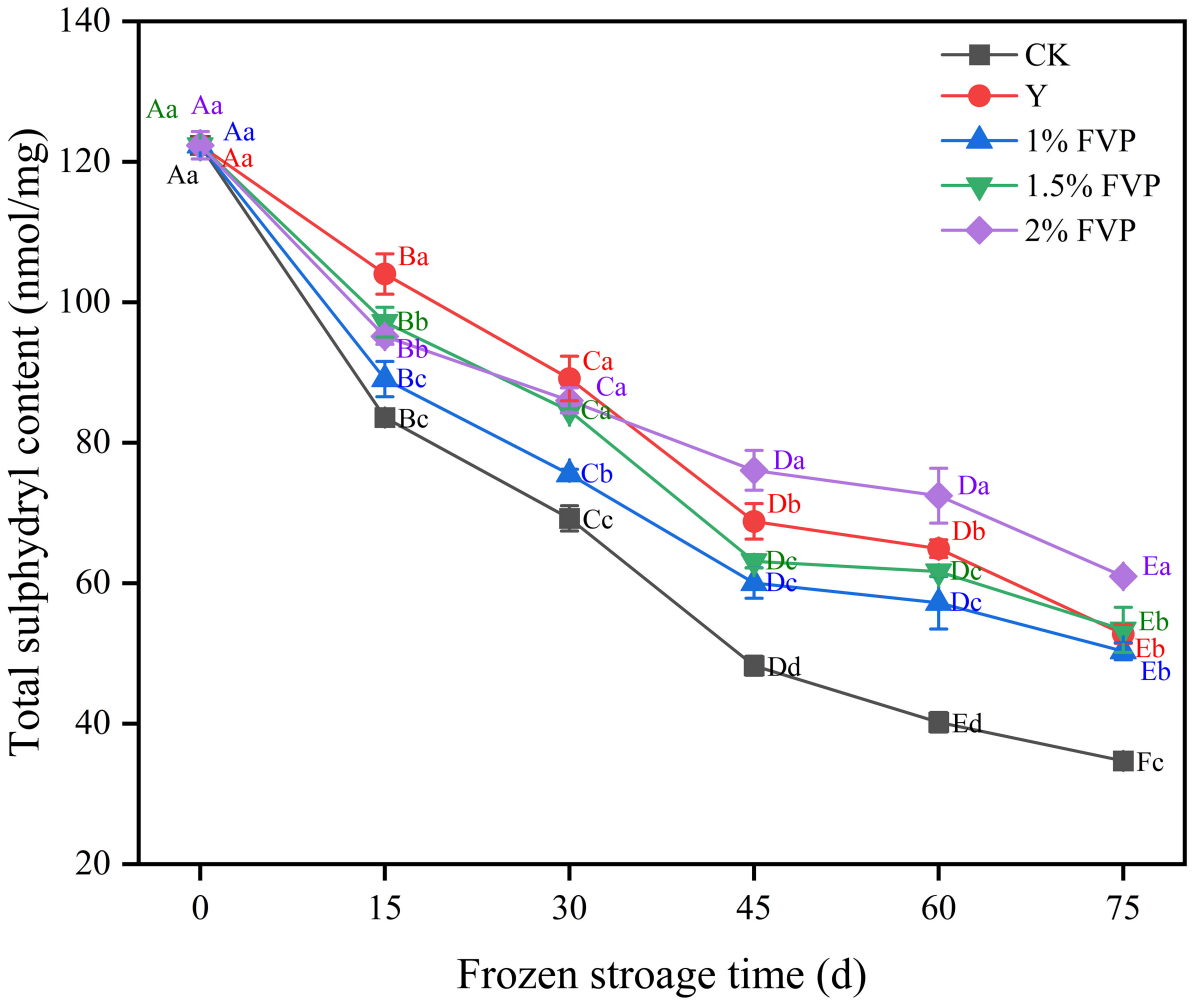
Effects of FVP on the sulfhydryl content of surimi MP. Data are expressed as mean ± standard deviation. Different lowercase letters indicated significant differences between different groups in the same freezing point (*P* < 0.05). Different capital letters indicated significant differences between different freezing sites in the same experimental group (*P* < 0.05).

The sulfhydryl is one of the most reactive functional groups in proteins, and can stabilize the spatial structure of MP (28). The effect of different cryoprotectant treatments on sulfhydryl content is shown in Figure 1. Overall, the sulfhydryl content of all samples decreased with an increase in freezing storage time. These findings were consistent with those reported by Cai et al. (29). Under the condition of moderate oxidation of MP, the sulfhydryl of MP is converted into disulfide bonds during the frozen storage, thus resulting in a decrease in the total sulfhydryl content. After 75 days of frozen storage, the total sulfhydryl content of surimi in the 2% FVP group was the highest (60.99 nmol/mg) followed by the 1.5% FVP and 1% FVP groups which were 53.37 nmol/mg and 50.33 nmol/mg, respectively. All these concentrations were significantly higher than those in the CK group (34.72 nmol/mg) (*P* < 0.05).

### 3.2. Effect of FVP on the carbonyl content of surimi MP

The carbonyl content of surimi MP is produced by the direct oxidation of amino acid side chains (30), which is an important indicator of the oxidation of surimi MP. Chain groups on the amino acid side of MP are easy targets for oxygen radicals that break peptide bond, leading to an increase in the content of carbonyl compounds and their derivatives.

**Figure 2:**
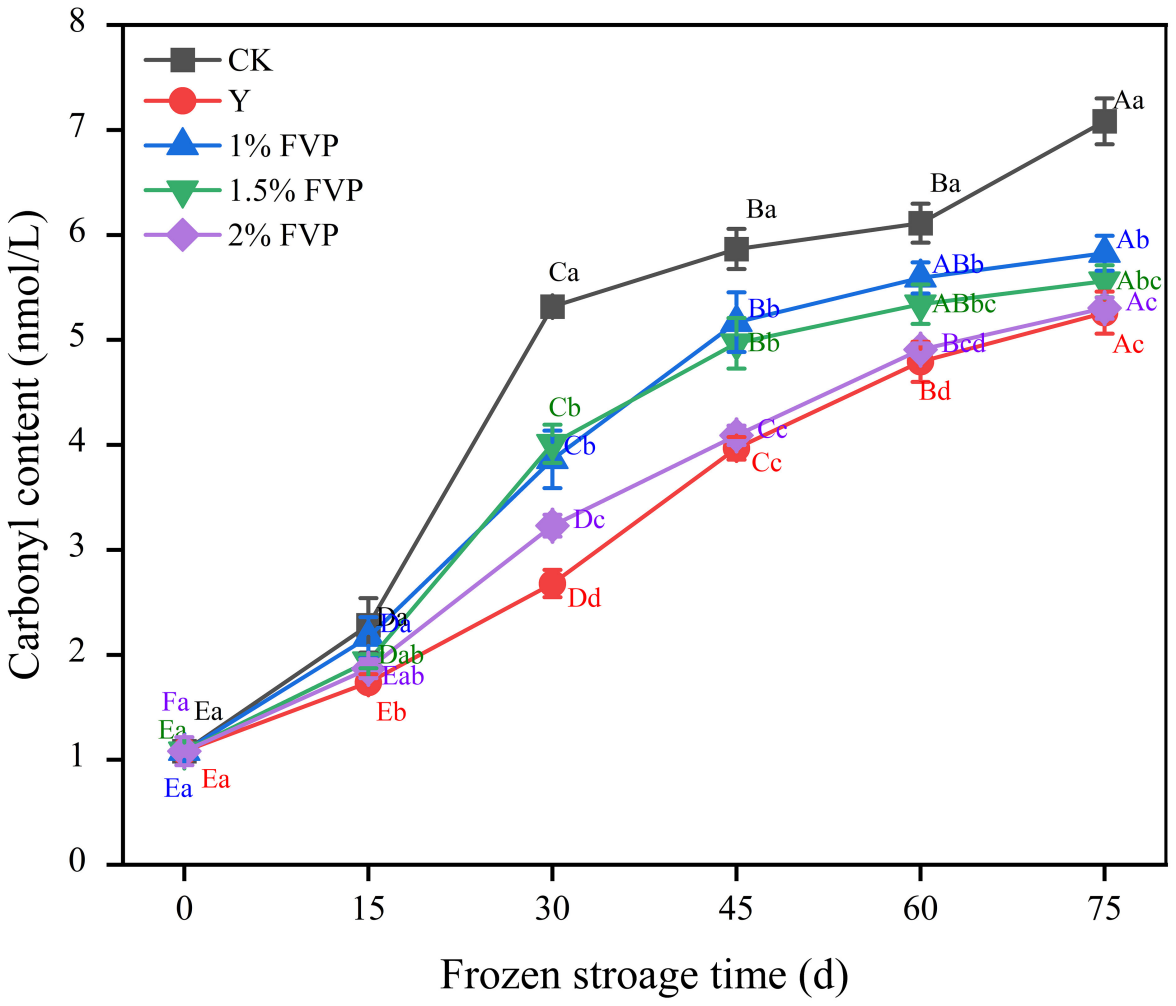
Effects of FVP on the carbonyl content of surimi MP. Data are expressed as mean ± standard deviation. Different lowercase letters indicated significant differences between different groups in the same freezing point (*P* < 0.05). Different capital letters indicated significant differences between different freezing sites in the same experimental group (*P* < 0.05).

As shown in Figure 2, the carbonyl content of surimi MP increased in all groups as the frozen storage time extended, which was consistent with the results of Zhang et al. (31). This suggested that frozen storage enhanced the degree of protein oxidation and increased the protein carbonyl content, which may be related to the release of oxidase and prooxidants from ruptured organelles (32). Throughout the storage process, the MP carbonyl content of surimi in the CK group increased by 6.00 nmol/L, which was significantly higher than the FVP groups, and the MP carbonyl content of surimi in the 1%, 1.5%, and 2% FVP groups and the Y group increased by 4.17–4.74 nmol/L. The greater the concentration of FVP, the lower the content of carbonyl. However, the differences in carbonyl content between the Y group and the 1.5% and 2% FVP groups were not significant (*P* > 0.05).

### 3.3. Effect of FVP on the solubility of surimi MP

**Figure 3:**
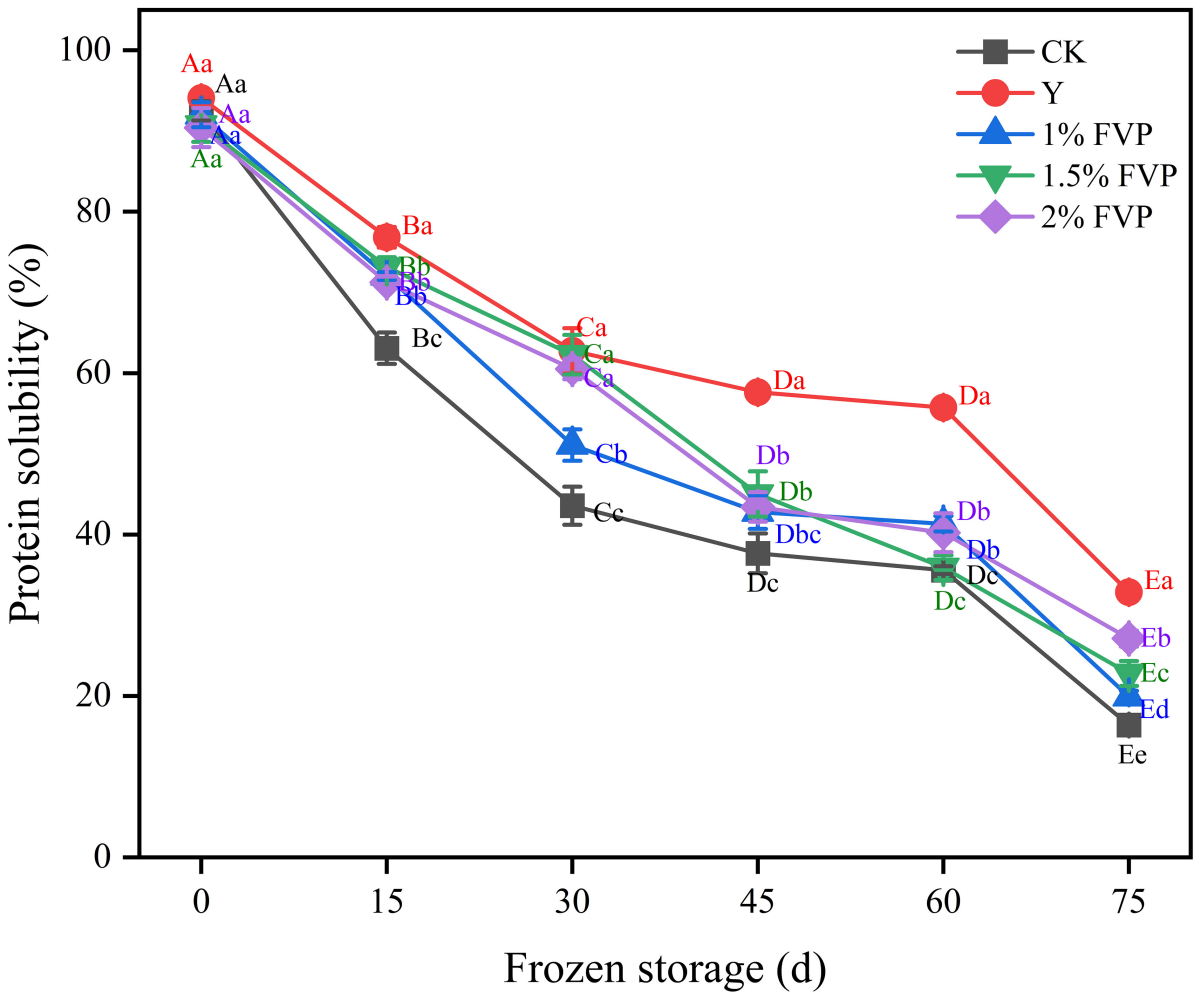
Effects of FVP on the solubility of surimi MP. Data are expressed as mean ± standard deviation. Different lowercase letters indicated significant differences between different groups in the same freezing point (*P* < 0.05). Different capital letters indicated significant differences between different freezing sites in the same experimental group (*P* < 0.05).

Protein solubility is a primary indicator of freezing-induced protein denaturation, which generally decreases due to hydrophobic interactions, hydrogen bonding, disulfide bonds, and ionic interactions during the frozen storage period (33). The effect of FVP on the solubility of surimi MP is shown in Figure 3, where the MP solubility of surimi decreased significantly (P < 0.05) in all groups before 30 days of frozen storage, thus indicating that a greater degree of MP denaturation occurs in the initial frozen period. These findings are in concordance with the previous studies ^[5]^. At the end of frozen storage, the MP solubility of surimi decreased by 76.10%, 61.23%, 72.10%, 67.95%, and 63.31% in the CK, Y, 1%, 1.5%, and 2% FVP groups, respectively. MP solubility of surimi in the CK group was significantly lower (*P* < 0.05) than in the Y and FVP groups implying that FVP can effectively mitigate the decrease in protein solubility. On the other hand, hydroxyl groups can bind free water molecules and convert them into binding water, thus preventing the formation of ice. This conversion can result in fewer ice crystals and weaken the protein denaturation caused by the formation of ice crystals (34).

### 3.4. Effect of FVP on Ca^2+^-ATPase activity in surimi

**Figure 4:**
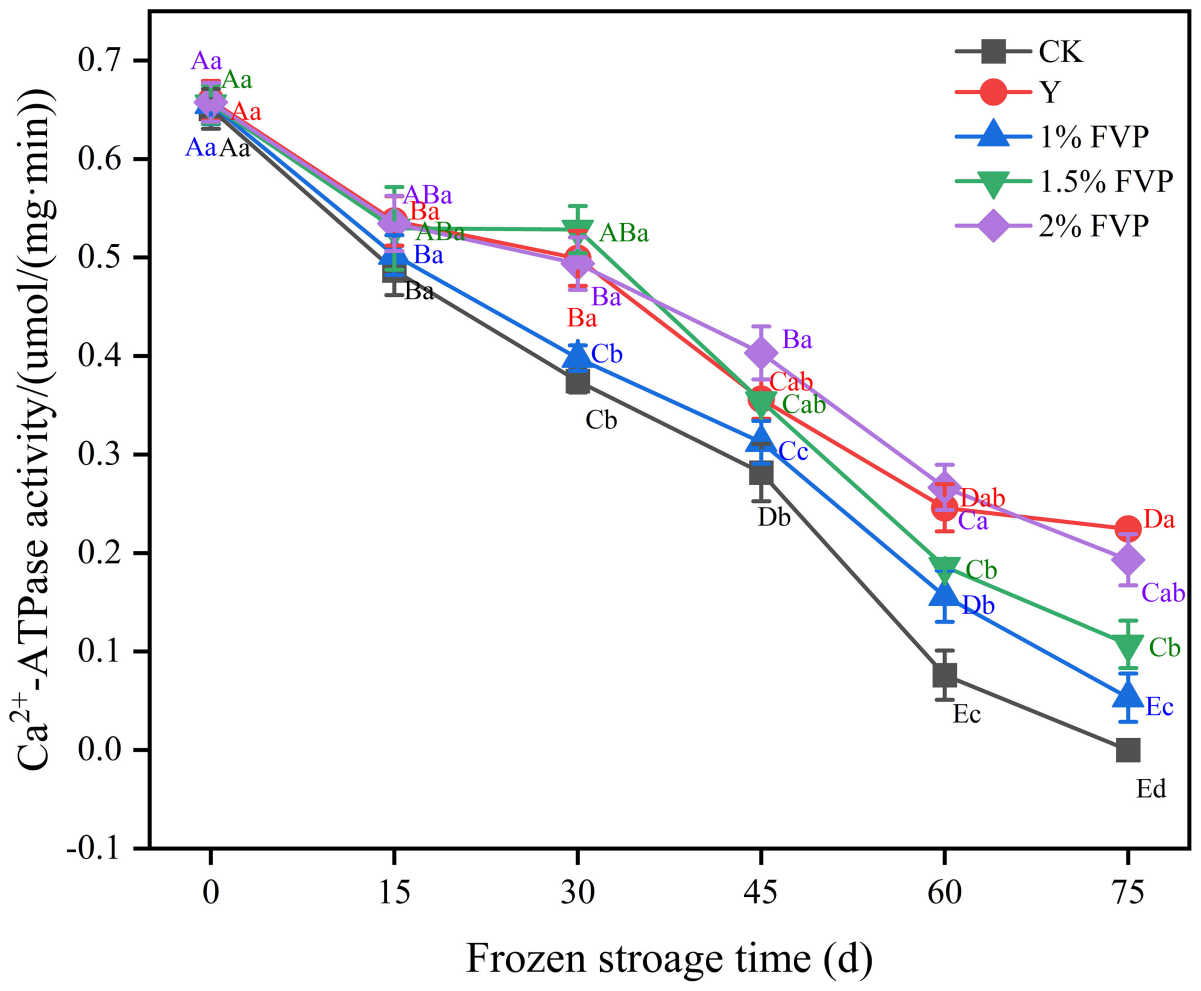
Effects of FVP on the Ca^2+^-ATPase activity of surimi MP. Data are expressed as mean ± standard deviation. Different lowercase letters indicated significant differences between different groups in the same freezing point (*P* < 0.05). Different capital letters indicated significant differences between different freezing sites in the same experimental group (*P* < 0.05).

Ca^2+^-ATPase activity is used as an indicator of myosin integrity and is widely used in the determination of the quality of frozen surimi (35). The Ca^2+^-ATPase activity of fresh surimi was 0.65 μmol/(mg min) and decreased continuously as the frozen storage time progressed, as shown in Figure 4. The Ca^2+^-ATPase activity of surimi in the CK group was lower than that of other experimental groups (Figure 4). A similar trend of decreased Ca^2+^-ATPase activity throughout frozen storage was also reported previously (36). At the end of frozen storage, Ca^2+^-ATPase activity was the highest in the Y group [0.22 μmol/(mg min) followed by the 2% FVP group (0.19 μmol/(mg min)], the 1.5% FVP group [0.11 μmol/(mg min)], and the 1% FVP group [0.05 μmol/(mg min)], and was the lowest in the CK group [0 μmol/(mg min)]. During frozen storage, the ice crystals change the structure of surimi proteins, resulting in a decrease in Ca^2+^-ATPase activity.

### 3.5. Effect of FVP on the water-holding capacity of surimi

**Figure 5:**
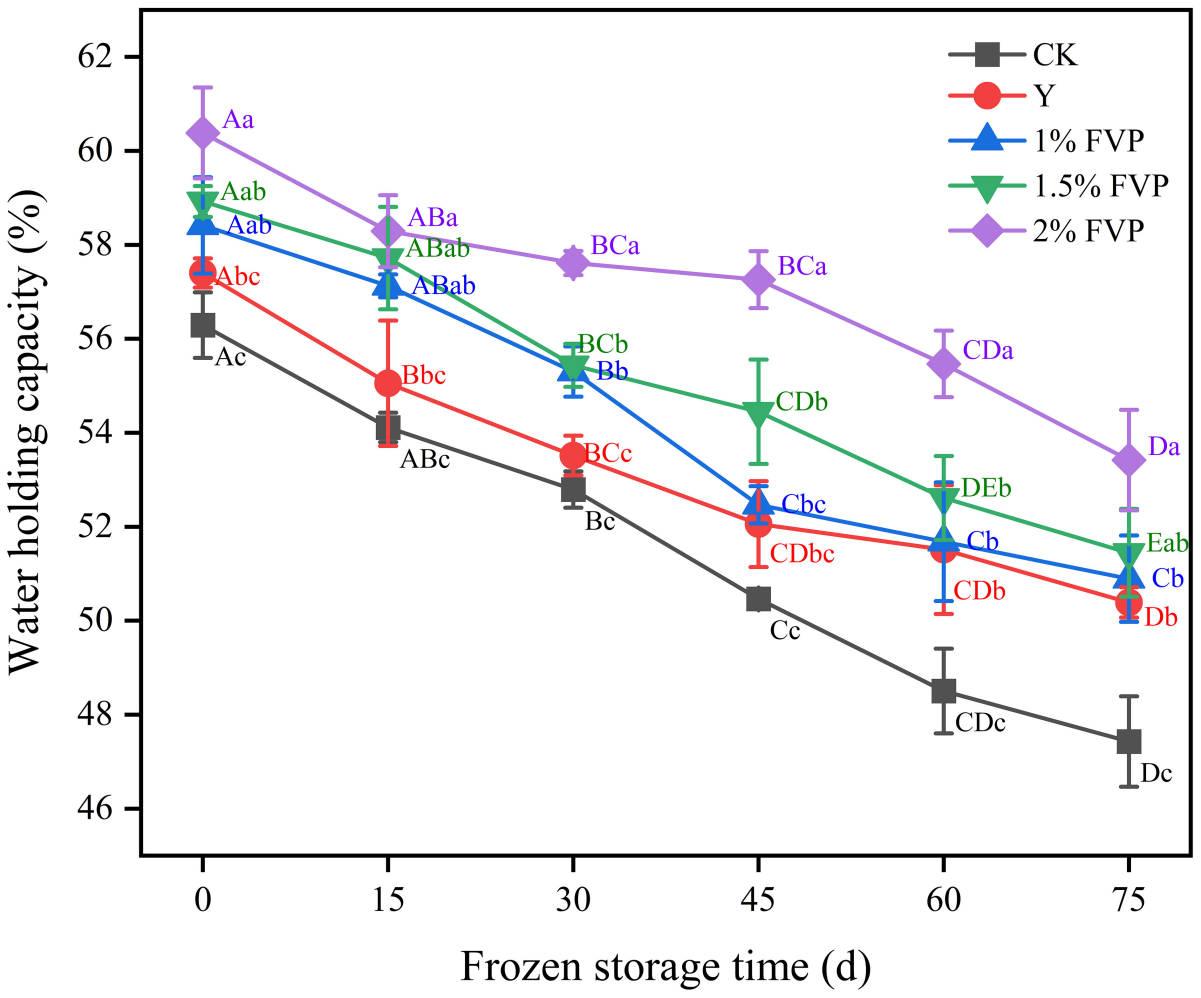
Effect of FVP on the water-holding capacity of surimi. Data are expressed as mean ± standard deviation. Different lowercase letters indicated significant differences between different groups in the same freezing point (*P* < 0.05). Different capital letters indicated significant differences between different freezing sites in the same experimental group (*P* < 0.05).

Water-holding capacity (WHC) is the ability to retain the original moisture content and add water when surimi suffers external forces that can visually evaluate the quality of surimi (37). As shown in Figure 5, the WHC of fresh surimi was 56.29% in the CK group and increased significantly with the addition of FVP (*P* < 0.05). Since FVP is hygroscopic, it can retain water content that can cross-link with surimi MP to become a stable complex (38). This is consistent with the results of Gao et al. (39), in which freezing storage led to the destruction of the secondary bonds that stabilize the MP structure. At 75 days of frozen storage, the WHC of surimi containing FVP was higher than that of the Y and CK groups, and the highest WHC was 53.42% in the 2% FVP group, followed by the 1.5% FVP group (51.44%). The lowest WHC was 47.43% in the CK group, which decreased by 8.86% compared with the fresh surimi. However, compared to the fresh fish surimi, the WHC of the 1%, 1.5%, and 2% FVP groups decreased by 7.52%, 6.48%, and 6.94%, respectively, thereby indicating that the addition of FVP can slow down the decrease of WHC in surimi during frozen storage.

### 3.6. Effect of FVP on color and pH of surimi

The color of surimi is determined by both the myoglobin content and the color of the additives (40), and white is more popular among consumers. Different levels of FVP affected the color values (L*, a*, b*, and whiteness) of surimi during 75-day storage (Table 1). Overall, adding FVP results in a significant decrease in the L^*^ and whiteness values of surimi compared with CK (*P* < 0.05). These changes were due to the yellowish color of FVP itself. However, the L^*^, a^*^, and b^*^ values of surimi decreased after adding the commercial protectants, which also led to a decrease in whiteness values. As storage time was prolonged, the whiteness of surimi decreased gradually. At the end of frozen storage, the whiteness value of surimi dropped to 41.98 and 39.10 in the CK and Y groups, respectively, and the FVP group had a lower level (32.98–35.97).

**Table 1:** Effect of FVP on the color and pH of surimi.

**Frozen storage time (day)**	**Group**	*L* ^*^	*a* ^*^	*b* ^*^	**Whiteness**	**pH**
0	CK	47.81 ± 0.87^Aa^	1.99 ± 0.52^Ac^	13.84 ± 0.75^ABb^	45.96 ± 0.80^Aa^	6.44 ± 0.02^Ba^
	Y	41.70 ± 1.07^ABb^	1.23 ± 0.15^BCd^	12.76 ± 0.53^Ab^	40.30 ± 0.99^ABb^	6.42 ± 0.02^Bab^
	1% FVP	41.40 ± 1.31^BCb^	3.50 ± 0.23^Ab^	17.17 ± 0.85^Aa^	38.83 ± 1.26^Bbc^	6.43 ± 0.03^Ca^
	1.5% FVP	39.84 ± 1.59^ABCbc^	4.02 ± 0.67^Aab^	17.01 ± 1.83^Aa^	37.33 ± 1.70^ABcd^	6.43 ± 0.02^Cab^
	2% FVP	38.33 ± 0.85^Bc^	4.63 ± 0.30^Aa^	18.77 ± 1.36^Aa^	35.36 ± 0.88^Bd^	6.37 ± 0.01^Cb^
15	CK	47.54 ± 0.86^Aa^	1.33 ± 0.18^Bb^	14.35 ± 0.73^Abc^	45.60 ± 0.91^Aa^	6.39 ± 0.08^Ba^
	Y	42.19 ± 0.78^ABc^	1.96 ± 0.18^Ab^	12.16 ± 0.78^Ac^	40.88 ± 0.65^ABb^	6.42 ± 0.00^Ba^
	1% FVP	44.64 ± 1.33^Ab^	3.52 ± 0.6^Aa^	17.23 ± 1.18^Aa^	41.91 ± 1.61^Ab^	6.44 ± 0.01^Ca^
	1.5% FVP	38.85 ± 1.84^BCd^	3.73 ± 0.95^Aa^	15.84 ± 2.64^Aab^	36.68 ± 2.38^ABc^	6.44 ± 0.00^Ca^
	2% FVP	40.09 ± 0.07^Ad^	4.01 ± 0.36^ABa^	17.70 ± 1.28^ABa^	37.39 ± 0.73^Ac^	6.42 ± 0.02^Ca^
30	CK	47.46 ± 1.01^Aa^	1.42 ± 0.13^Bc^	14.42 ± 0.56^Ab^	45.49 ± 0.86^ABa^	6.62 ± 0.02^Aa^
	Y	43.25 ± 2.72^Ab^	1.62 ± 0.37^ABc^	12.04 ± 1.57^Ac^	41.95 ± 2.65^ABb^	6.74 ± 0.01^Aa^
	1% FVP	42.98 ± 1.27^ABb^	3.03 ± 0.37^ABb^	16.74 ± 1.32^Aa^	40.49 ± 1.42^ABbc^	6.62 ± 0.01^Ba^
	1.5% FVP	41.26 ± 1.02^ABbc^	3.56 ± 0.23^ABb^	17.25 ± 0.91^Aa^	38.67 ± 1.01^Ac^	6.62 ± 0.05^Ba^
	2% FVP	38.84 ± 0.70^ABc^	4.15 ± 0.39^ABa^	17.91 ± 1.21^ABa^	36.12 ± 0.52^ABd^	6.60 ± 0.02^ABa^
45	CK	45.64 ± 1.07^Ba^	1.36 ± 0.24^Bc^	13.82 ± 1.16^ABbc^	43.88 ± 1.21^BCa^	6.63 ± 0.02^Aa^
	Y	43.16 ± 0.75^Aa^	0.72 ± 0.42^Cd^	11.21 ± 1.96^ABc^	42.04 ± 1.75^ABa^	6.63 ± 0.01^Aa^
	1% FVP	40.36 ± 2.54^Cb^	2.56 ± 0.46^Bb^	14.16 ± 1.67^Bb^	38.62 ± 1.83^Bb^	6.60 ± 0.01^Ba^
	1.5% FVP	39.54 ± 3.91^ABCbc^	3.48 ± 0.26^ABa^	17.65 ± 2.08^Aa^	36.89 ± 1.31^ABbc^	6.61 ± 0.02^Ba^
	2% FVP	37.50 ± 3.94^Bc^	3.89 ± 0.41^Ba^	17.33 ± 1.10^ABa^	35.02 ± 1.23^Bc^	6.57 ± 0.02^Ba^
60	CK	45.00 ± 1.26^Ba^	1.25 ± 0.22^Bc^	13.16 ± 0.76^ABc^	43.42 ± 1.18^CDa^	6.68 ± 0.05^Aa^
	Y	43.17 ± 1.66^Aab^	1.05 ± 0.27^BCc^	9.52 ± 1.30^Bd^	42.36 ± 1.85^Aa^	6.66 ± 0.03^Aa^
	1% FVP	41.54 ± 0.79^BCb^	2.64 ± 0.26^Bb^	14.99 ± 1.01^ABbc^	39.59 ± 0.98^ABb^	6.69 ± 0.00^Aa^
	1.5% FVP	41.51 ± 1.82^Ab^	2.55 ± 0.69^Bb^	15.76 ± 1.68^Aab^	39.34 ± 1.78^Ab^	6.67 ± 0.01^Aa^
	2% FVP	38.30 ± 0.70^Bc^	3.58 ± 0.42^Ba^	17.34 ± 0.55^ABa^	35.80 ± 0.76^Bc^	6.60 ± 0.01^ABb^
75	CK	43.34 ± 0.25^Ca^	1.22 ± 0.30^Bc^	12.32 ± 1.35^Bb^	41.98 ± 0.23^Da^	6.74 ± 0.02^Aab^
	Y	40.12 ± 1.67^Bb^	0.82 ± 0.45^Cc^	11.05 ± 0.64^ABb^	39.10 ± 1.70^Bb^	6.78 ± 0.00^Aa^
	1% FVP	38.10 ± 0.55^Dc^	3.21 ± 0.25^ABb^	15.94 ± 1.98^ABa^	35.97 ± 0.64^Cc^	6.70 ± 0.02^Abc^
	1.5% FVP	37.31 ± 1.14^Cc^	3.68 ± 0.33^Aab^	17.11 ± 0.49^Aa^	34.91 ± 1.18^Bc^	6.70 ± 0.01^Abc^
	2% FVP	35.18 ± 1.15^Cd^	4.03 ± 0.22^ABa^	16.49 ± 1.67^Ba^	32.98 ± 0.76^Cd^	6.67 ± 0.03^Ac^

Data are expressed as mean ± standard deviation. Different lowercase letters indicated significant differences between different groups in the same freezing point (*P* < 0.05). Different capital letters indicated significant differences between different freezing sites in the same experimental group (*P* < 0.05).

A series of biochemical reactions in the surimi can lead to a change in pH during frozen storage, so the quality of the surimi can be estimated by measuring the change in pH (41). From Table 1, the pH of fresh surimi was 6.44, and the pH of FVP-added surimi decreased. This is due to the fact that the pH of FVP is lower than that of surimi itself. The pH of surimi showed an increasing trend during frozen storage, probably because of the degradation of proteins and production of alkaline substances such as amines (42), which is consistent with the trend of pH changes during freeze–thaw cycles of surimi products studied by Wilayat et al. (43). At 75 days of frozen storage, the pH of the CK group was lower than that of the FVP groups significantly (*P* < 0.05).

### 3.7. Effect of FVP on the gel strength of surimi

**Figure 6:**
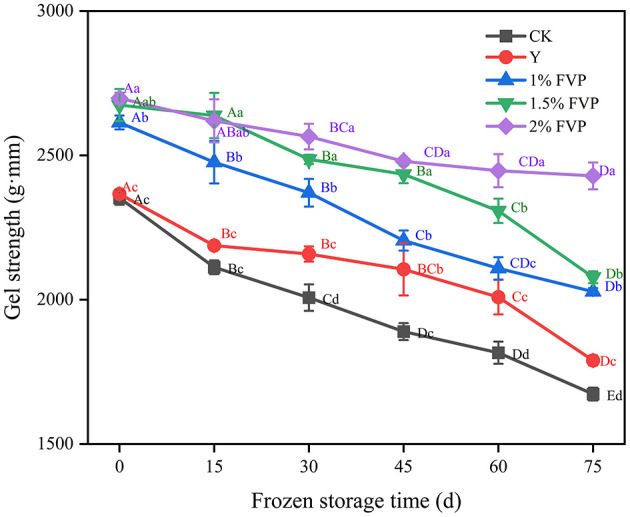
Effect of FVP on the gel strength of surimi. Data are expressed as mean ± standard deviation. Different lowercase letters indicated significant differences between different groups in the same freezing point (*P* < 0.05). Different capital letters indicated significant differences between different freezing sites in the same experimental group (*P* < 0.05).

Gel strength is an essential index to evaluate the quality of surimi products, with higher gel strength values indicating better quality surimi products (44). From Figure 6, the gel strength of fresh surimi was 2352.87 g·mm, and the effect of adding commercial protectant (Y group) on the gel strength of surimi was not significant (*P* > 0.05), while the gel strength of FVP-added surimi increased significantly (*P* < 0.05). FVP is easily expanded by heat after absorbing water and fills in the gaps of the gel network structure of surimi, making the gel structure of surimi denser (45). The expanded FVP would exert a certain pressure on the surimi protein, which can further strengthen the gel strength of the surimi. The gel strength of surimi in the CK group was lower than that in the Y and FVP groups throughout the frozen period, and at 75 days of frozen storage, the maximum gel strength of surimi was 2428.92 g·mm in the 2% FVP group, which was 755.93 g·mm higher than that of the CK group.

### 3.8. Effect of FVP on the rheological properties of surimi

**Figure 7:**
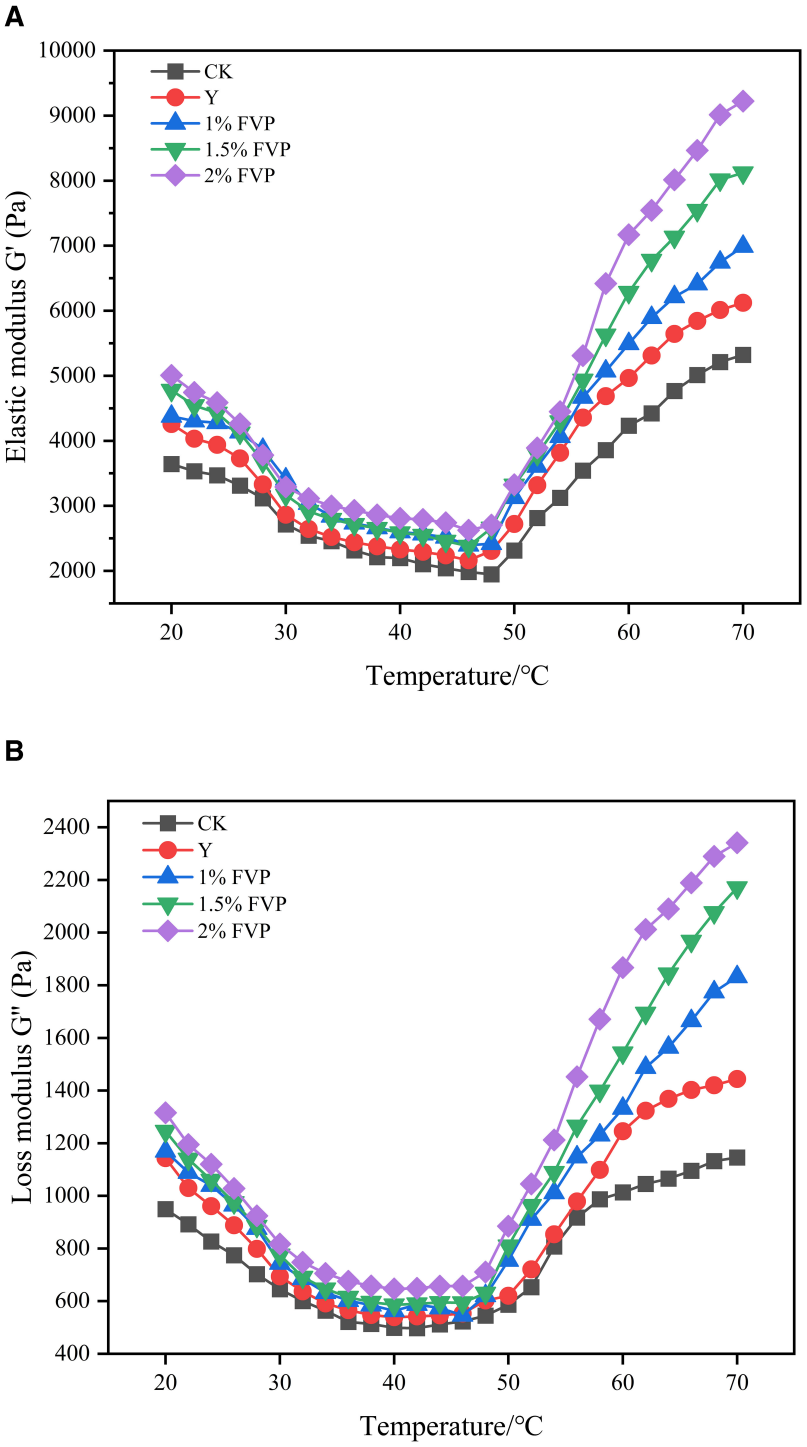
Effect of FVP on the energy storage modulus (G') **(A)** and the loss modulus (G”) **(B)** of surimi. Data are expressed as mean ± standard deviation. Different lowercase letters indicated significant differences between different groups in the same freezing point (*P* < 0.05). Different capital letters indicated significant differences between different freezing sites in the same experimental group (*P* < 0.05).

The energy storage modulus (G') is known as the elastic modulus, which reflects the formation of the protein gel network structure. The loss modulus (G”) is known as the viscous modulus, which reflects the viscous characteristics of the samples (46). Figure 7 shows the changes in G' and G” during the temperature scan from 20 to 70°C with different experimental groups. The responses for all samples follow a similar trend in which two stages can be distinguished. In the first stage, G′ and G″ decreased with temperature increasing to a minimum of ~50°C because the temperature at this point is the optimal activity temperature of endogenous hydrolase, the network structure of actin and myosin is broken, and some chemical bonds between protein molecules are broken, which makes protein molecules extend and surimi enter the gel deterioration stage. In the second stage, the changes, including the gel networks, hydrophobic bonds, disulfide bonds, and other chemical bonds, and the three-dimensional gel structure occurred (47), so that G′ and G″ increased rapidly to their maximum of ~70°C. G' and G” of the CK group were lower than those of the Y and FVP groups throughout the frozen process, which indicated that FVP and commercial protectants had a protective effect on the gel network structure of surimi. The FVP molecules improve the gel properties by filling the gaps of proteins and interacting with them, resulting in greater viscoelasticity when the FVP is added to the surimi. The best gel properties were found in the surimi of the 2% FVP group.

### 3.9. Effect of FVP on the TBARS value of surimi

**Figure 8:**
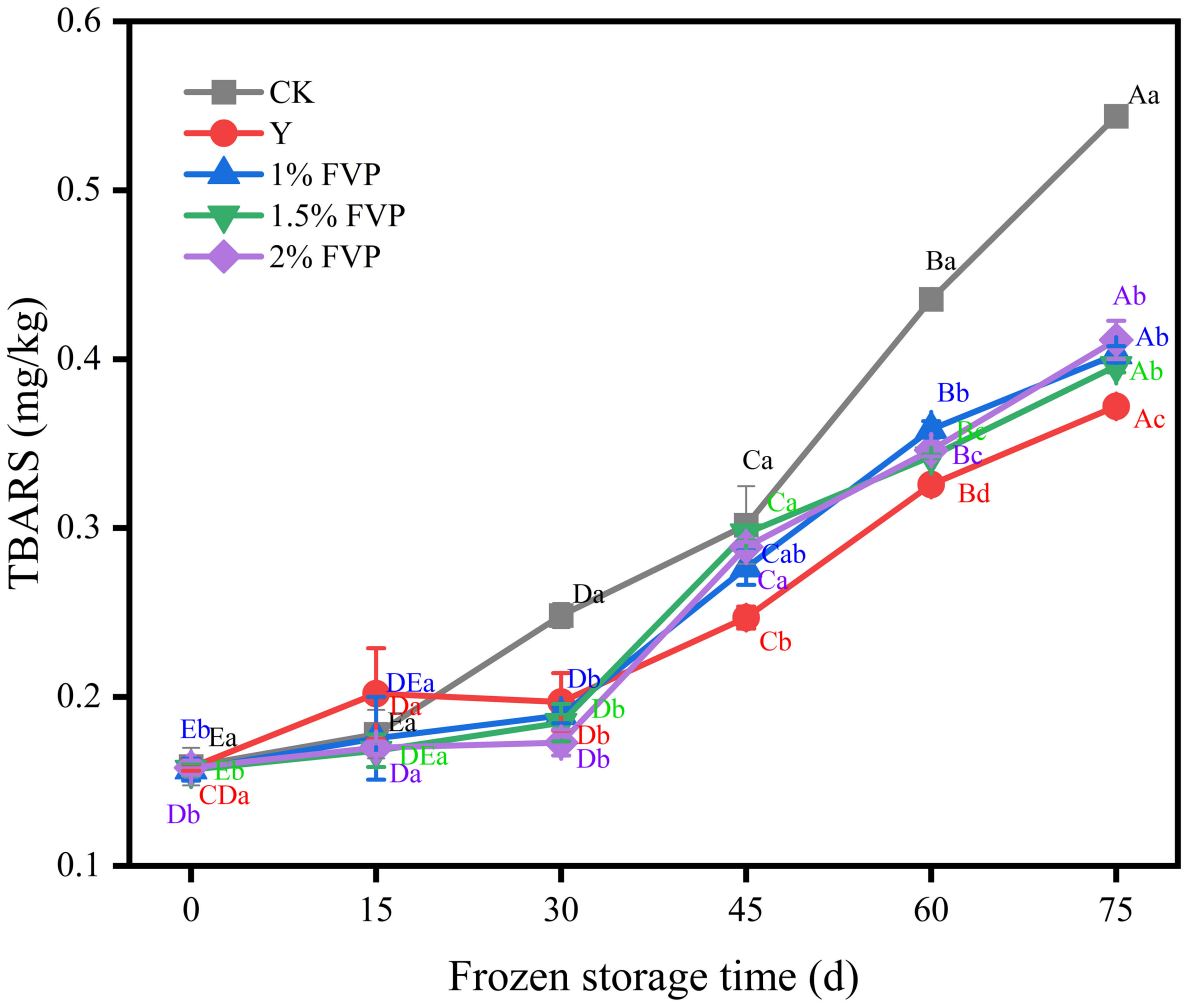
Effect of FVP on the TBARS of surimi. Data are expressed as mean ± standard deviation. Different lowercase letters indicated significant differences between different groups in the same freezing point (*P* < 0.05). Different capital letters indicated significant differences between different freezing sites in the same experimental group (*P* < 0.05).

Lipid oxidation produces complex reaction products such as ketones, aldehydes, hydrocarbons, and esters, which lead to off-flavors in surimi and negatively affect its quality (48). TBARS is widely used as an indicator to assess the degree of lipid oxidation (11). As shown in Figure 8, although the TBARS values increased slowly from 0 to 30 days of frozen storage, the TBARS values in all groups showed an increasing trend with the extension of frozen time. The CK groups were always higher than the FVP groups during the whole frozen storage time. After 75 days of frozen storage, the TBARS values of surimi in the CK group increased from 0.16 mg/kg to 0.54 mg/kg, an increase of 0.38 mg/kg, and the Y group was the lowest, only increasing by 0.17 mg/kg. When supplemented with 1%, 1.5%, and 2% FVP, the TBARS values increased by 0.25 mg/kg, 0.24 mg/kg, and 0.23 mg/kg, respectively, all of which were lower than the CK group. These results show that FVP exerts an inhibitory effect on the lipid oxidation of surimi (20). However, with the extension of frozen time, the antioxidant capacity of FVP diminishes, leading to a significant increase in the degree of oxidation of surimi in the later stages of freezing (*P* < 0.05).

### 3.10. Effect of FVP on the microstructure of surimi

**Figure 9:**
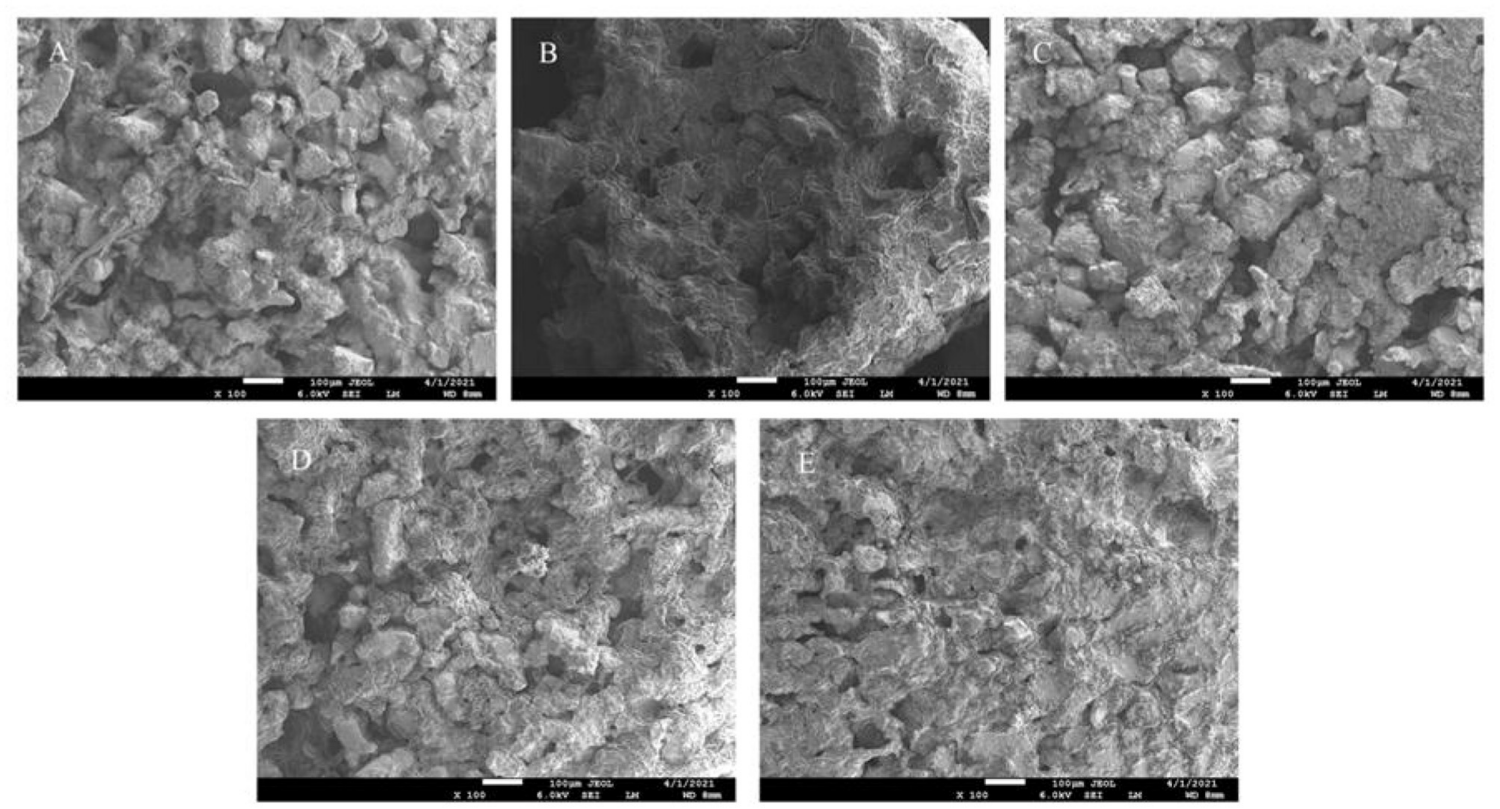
SEM photographs of CK group **(A)**, Y group **(B)**, 1% FVP group **(C)**, 1.5% FVP group **(D)**, and 2% FVP group **(E)**. Data are expressed as mean ± standard deviation. Different lowercase letters indicated significant differences between different groups in the same freezing point (*P* < 0.05). Different capital letters indicated significant differences between different freezing sites in the same experimental group (*P* < 0.05).

The microstructures of surimi with different levels of FVP were visualized by SEM, as shown in Figure 9. There were more pores with larger pore sizes in the CK group, and the overall structure was rough and loose after 75 days of frozen storage. However, with the addition of FVP at 2%, the surface of the surimi gel was flat and smooth, and the network structure was tight, which was not significantly different from that of the Y group. Therefore, the pores of surimi gels in the CK group may be formed by water loss, while FVP can closely link with proteins to improve the gel quality. In addition, the multi-OH structure of FVP itself can absorb water lost during the thermal denaturation of surimi proteins to improve WHC and reduce pores. Zhuang et al. (49) also found that polysaccharides were beneficial for improving the structural properties of the composite gel.

## 4. Discussion

*Flammulina velutipes* is a popular mushroom species and has long been known for its nutritive and medicinal properties in folk medicine. While it is edible, the roots of *Flammulina velutipes* are often discarded due to difficulty in chewing, resulting in a waste of resources. However, the roots are rich in FVP, the most active ingredient found in *Flammulina velutipes*. In the current study, FVP was extracted from the roots of *Flammulina velutipes* using ultrasound-assisted hot water extraction. The molecular weight of FVP was found to be in the range of 27 KDa−9,178 KDa and mainly consists of glucose (Glc), galactose (Gal), mannose (Man), fucose (Fuc), and xylose (Xyl) molecules linked by α-1,4- and α-1,6-glycosidic chains.

MP is an important component of myofibrils, and ~85% of water is retained in the MP structure. Research evidence has shown that freezing causes disruption of secondary bonds that stabilize the MP structure. Thus, the hydrophobic groups of MP are exposed and interact with adjacent protein regions, resulting in MP aggregation denaturation and decreased water retention. As a macromolecule polysaccharide, FVP may also form a glassy system, in which the water in the tissues reaches the freezing concentration state maximally, and the migration rate of water molecules is greatly reduced, which can effectively avoid aggregation denaturation and ice recrystallization (50).

The gel strength can well reflect the ability of proteins to form a gel, which is a primary index to evaluate the quality of surimi products. During the frozen storage process, trimethylamine demethylase is released into muscle tissue, then oxidizes trimethylamine to form formaldehyde, which always destroys the integrity of myosin (51), thus leading to the decrease of gel ability of surimi protein. On the other hand, the oxidation of fat during frozen storage also adversely affects the structure and function of surimi protein, leading to a decrease in surimi gel strength. The hydroxyl groups in the FVP can replace the water binding site and form strong hydrogen bonds with the polar residues of protein molecules to stabilize the protein structure of surimi, thus effectively improving the gel strength of surimi and slowing down the decrease in gel strength during frozen storage.

The FVP is easily expanded by heat after absorbing water and then fills in the gaps of the gel network structure in surimi, making the surimi gel structure denser. The expanded FVP will exert a certain pressure on the surimi protein, and the surimi network structure will become stronger. Electron microscopy images showed that the microstructure of surimi gel had a flat and smooth surface and a tight network structure, which was because FVP molecules would improve the gel properties by filling gaps between proteins and interacting with proteins to improve the compactness and denseness of the gel structure.

## 5. Conclusion

The research suggests that, as a novel cryoprotectant, FVP can significantly enhance the quality of surimi products while avoiding an excessively sweet taste and high-calorie value. Subsequently, the frozen storage of surimi indicated that adding FVP could alleviate the decline in MP solubility, Ca^2+^-ATPase activity, total sulfhydryl content, and water-holding capacity of catfish surimi. In addition, the addition of FVP could inhibit the increase in TBARS values, improve the rheological properties of surimi gel, and enhance the gel strength of surimi, and the microstructure showed that the gel structure of surimi with the addition of FVP was denser. Using the above indicators, 2% FVP effectively mitigated the protein denaturation of catfish surimi during frozen storage. Therefore, FVP is expected to be used as a new type of cryoprotectant in the frozen storage of surimi products.

## Data availability statement

The raw data supporting the conclusions of this article will be made available by the authors, without undue reservation.

## Author contributions

LL: Conceptualization, Data curation, Resources, Validation, Writing—original draft, Writing—review and editing. YL: Conceptualization, Writing—review and editing. XZ: Investigation, Writing—review and editing. TA: Writing—review and editing. MS: Writing—review and editing. MYS: Writing—review and editing. YW: Writing—review and editing. CC: Writing—review and editing. YZ: Funding acquisition, Project administration, Supervision, Writing—review and editing.
